# Preoperative 3D-Planned S1 Corridors Transferred into 2D Fluoroscopy Allow for Safe Intraoperative Large-Diameter Implant Placement: Description of a Novel Sacroiliac Fixation Technique and Proof of Concept in 137 Implantations

**DOI:** 10.3390/medicina62061100

**Published:** 2026-06-05

**Authors:** Frederic Bludau, Steffen Heinrich Schulz, Sascha Gravius, Peter Fennema, Marcus Rickert, Johannes Vogel, Franz-Joseph Dally

**Affiliations:** 1 Orthopaedic and Trauma Surgery Center, University Medical Center Mannheim, University of Heidelberg, Theodor-Kutzer-Ufer 1-3, 68167 Mannheim, Germany; frederic.bludau@umm.de (F.B.); sascha.gravius@umm.de (S.G.); johannes.vogel@umm.de (J.V.); franz.dally@umm.de (F.-J.D.); 2 AMR Advanced Medical Research, 8708 Männedorf, Switzerland; p.fennema@amr-cro.com; 3 Orthopädie Rickert, 63500 Seligenstadt, Germany; rickert@dr-marcus-rickert.de

**Keywords:** fragility fractures of the pelvis, sacral insufficiency fracture, iliosacral screw fixation, osteoporotic fractures, sacroiliac joint, imaging, three-dimensional, fluoroscopy, titanium

## Abstract

*Background and Objectives*: Percutaneous iliosacral screw fixation is a standard treatment for posterior pelvic ring instability and sacral insufficiency fractures. However, conventional transsacral S1 screw fixation is associated with notable complication rates, most commonly implant loosening; dysmorphic sacral anatomy increases the risk of iatrogenic L5 or S1 nerve root injury. This study presents a modified S1 trajectory to engage the high-density bone of the anterior and cranial S1 vertebral body (promontory) by transferring preoperative 3D planning to intraoperative 2D fluoroscopy. *Materials and Methods*: This retrospective study analyzed implant placements for posterior pelvic ring instability, including high-velocity trauma and fragility fractures of the pelvis (FFPs). Preoperative computed tomography (CT) multiplanar reconstruction defined a modified corridor from a posterior-caudal iliac entry point directed cranially and ventrally into the S1 promontory. The 3D trajectory was transferred intraoperatively using standard 2D fluoroscopy (lateral, anteroposterior, inlet, and outlet views) with the patient prone. In cases of reduced bone quality or intended sacroiliac fusion, 3D-printed titanium implants (triangular or cylindrical threaded, 10.0–13.5 mm outer diameter) were selected over 7.5 mm cannulated screws. *Results*: Overall, 137 implants were placed in 71 patients: 13 cannulated screws in high-velocity pelvic ring trauma, 72 triangular titanium sacroiliac fusion implants (iFuse Implant System^®^, SI-Bone), and 52 threaded titanium fusion implants (iFuse TORQ^®^, SI-Bone) in patients with FFP. The modified trajectory consistently engaged the anterior and cranial S1 vertebral body. Postoperative 3D CT confirmed accurate placement of all implants. No iatrogenic nerve injuries or revisions for implant malposition occurred. Mean follow-up was 12 ± 9 months. *Conclusions*: Preoperative 3D CT planning combined with standard 2D fluoroscopy guided a modified S1 trajectory toward the cranial S1 vertebral body. Accurate and safe implant placement was achieved in the prone position without navigation systems, providing a practical alternative when standard transverse trajectories are limited by narrow bony corridors or sacral or pelvic dysmorphy.

## 1. Introduction

The increasing incidence of fragility fractures of the pelvis (FFPs) amid demographic aging has created a “silent epidemic”, posing a substantial clinical challenge [1]. Historically, conservative management was the standard practice; however, the morbidity associated with sustained immobility has prompted a shift to surgical stabilization to facilitate early mobilization [2,3]. Percutaneous iliosacral screw fixation is widely accepted as a minimally invasive method for stabilizing the posterior pelvic ring [4,5]. However, in osteoporotic patients, this technique is associated with higher complication rates; specifically, inadequate implant anchorage results in implant loosening or backing out in up to 20% of cases [6,7].

The progressive nature of FFPs is relevant when indications for early surgical treatment are considered. Unlike high-energy trauma, FFPs may follow a dynamic process termed an “instability cascade” [8]. This model describes a gradual loss of pelvic stability that may begin with a non-displaced unilateral sacral ala fracture (FFP type II) [9]. Ongoing pain and antalgic gait can alter load transmission, increasing stress on the contralateral sacral ala and pubic rami [10]. In osteoporotic bone, this altered loading may cause additional insufficiency fractures, with progression to bilateral instability (FFP type III or IV) and, in advanced cases, spinopelvic dissociation [9]. We hypothesized that titanium sacroiliac fusion implants provide a larger surface contact area with increased rotational resistance than conventional screws do, which may limit micromotion associated with traditional screw toggling [11] and increase bony integration because of the macroscopically rough surface texture. These implants are geometrically designed to restrict rotation, thereby reducing further mechanical deterioration.

Effective stabilization is frequently limited by reduced bone quality in the geriatric pelvis. The risk of fixation failure in this population is affected by the trajectory used in standard techniques. Conventional fluoroscopic approaches, such as those described by Krappinger et al., typically target a transverse corridor through the S1 vertebral body [4]. Although it is suitable in non-osteoporotic bone, this trajectory often traverses the sacral ala [2]. Morphometric and densitometric analyses have identified the sacral ala as a region of lowest bone mineral density, commonly termed the “alar void” [2]. In osteoporotic sacra, this region exhibits poor resistance to screw toggling.

Improved biomechanical fixation requires engagement of regions with high bone mineral density. Quantitative analyses have demonstrated nonuniform distribution of bone density within the sacrum. Although the S1 ala exhibits reduced density in osteoporotic patients, the cranial and ventral portion of the S1 vertebral body, the promontory, retains comparatively higher density [2,12]. Accordingly, a biomechanically favorable trajectory for treating fragility fractures is oblique rather than transverse, originating from a more posterior and caudal iliac entry point and directed cranially and anteriorly toward the cranial S1 vertebral body.

Preoperative 3D planning based on multiplanar CT reconstruction has become an established tool in orthopaedic and pelvic trauma surgery, allowing complex fracture morphology to be analysed and surgical strategy to be defined before the procedure [13]. Atlas-based 3D planning has been combined with 2D fluoroscopic guidance to translate the preoperative plan into the operating room without dedicated navigation systems [14]. In the specific context of iliosacral fixation, preoperative CT simulation has been used to optimise screw trajectories and to avoid neurovascular structures [15]. The present study extends this approach by integrating density-based 3D planning with a modified S1 trajectory and translating the plan to standard 2D fluoroscopic guidance, without reliance on intraoperative navigation systems.

On the basis of these findings, the present study presents a surgical technique that incorporates density-based planning. Preoperative 3D multiplanar reconstruction was used to define a modified S1 corridor, which was subsequently transferred to intraoperative 2D fluoroscopy. Notably, the entire procedure is performed with the patient in the prone position, which facilitates posterior access to the caudal iliac entry point and optimizes fluoroscopic visualization of the sacral landmarks required for this modified trajectory. To address rotational instability associated with conventional screws, 3D-printed titanium implants—either triangular or cylindrical threaded up to 13.5 mm in diameter—are employed for sacroiliac fusion. In biomechanical testing, large-diameter triangular or cylindrical threaded implants have demonstrated significantly higher insertion and removal torques than standard 7.3 mm lag screws, indicating greater initial anchorage in the sacral bone [16].

## 2. Materials and Methods

### 2.1. Study Design and Patient Selection

**Figure 1:**
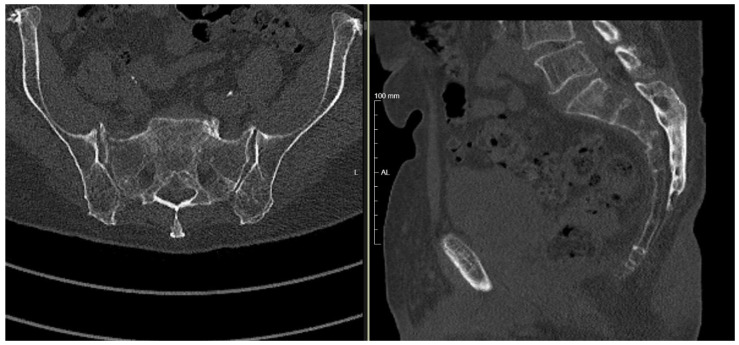
Preoperative CT imaging of an H-type sacral insufficiency fracture (FFP IVb). Axial (**left**) and sagittal (**right**) reconstructions demonstrate the fracture configuration of the posterior pelvic ring.

This study is a retrospective analysis of a consecutive series of patients treated for posterior pelvic ring instability between February 2021 and November 2025. The cohort comprised 137 implant placements (13 screws; 72 iFuse Implant System^®^, 52 iFuse TORQ^®^ SI-BONE Inc., Santa Clara, CA, USA) in patients with either high-velocity trauma or FFP. Indications for surgical stabilization included pelvic ring fractures, sacroiliac joint disruptions, sacral fractures (Denis types I–III), transalar or transforaminal fractures, and sacral insufficiency fracture patterns (FFP IIIb and c, FFP IVb; OF3; OF4 and sub-S2 H-type or U-type) in which stabilization was indicated because of intractable pain or failure of mobilization (Figure 1). Displaced fractures requiring open reduction and decompression were excluded.

### 2.2. Preoperative 3D Planning

**Figure 2:**
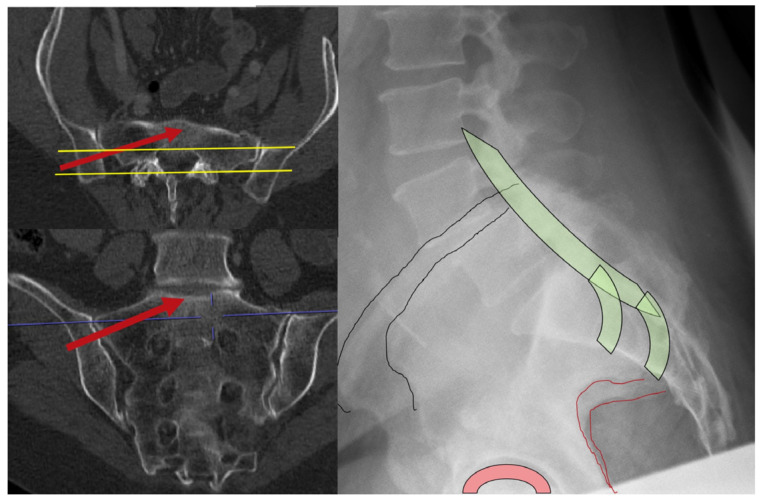
CT-based preoperative planning of the modified S1 corridor and correlation with the lateral projection. Multiplanar reconstructions and the corresponding lateral projection illustrate the planned implant corridor and the posterior-caudal entry point, which is defined on the lateral view relative to the posterior border of the spinal canal according to the preoperative CT plan.

**Figure 3:**
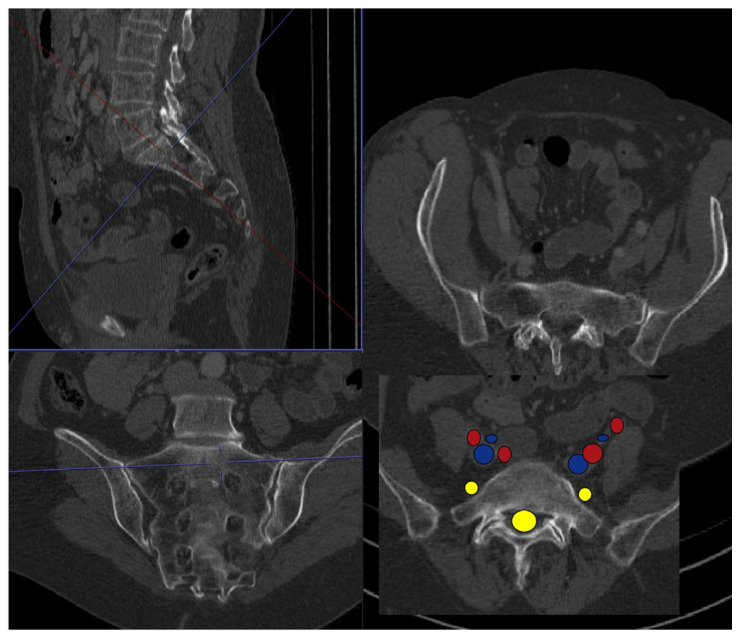
CT-based preoperative planning of the modified S1 corridor with anatomic safety mapping. Multiplanar CT reconstructions illustrate the planned implant corridor and its spatial relationship to the spinal canal, sacral neuroforamina, and adjacent neural and vascular structures.

All patients underwent preoperative computed tomography (CT) of the pelvis. Unlike standard transverse planning, this approach defined a modified S1 corridor targeting regions of higher bone mineral density, typically located in the cranial and ventral portion of the S1 vertebral body, specifically the promontory. The planned trajectory originated from a more posterior and caudal iliac entry point and extended obliquely toward the cranial-ventral S1 body (Figure 2). Multiplanar reconstructions aligned with the S1 superior endplate were then used to assess sacral morphology and measure corridor dimensions in order to determine the appropriate implant length and diameter while maintaining osseous containment within the sacral ala and vertebral body [2] (Figure 3). Preoperative CT studies were obtained at our institution on one of approximately fifteen scanners with different manufacturers and years; the tube voltage and tube current used followed each scanner’s standard pelvic protocol and therefore varied across the cohort, and all studies were reconstructed at a slice thickness of 1 mm. Multiplanar reconstruction and 3D corridor planning were performed using Syngo Carbon Enterprise Access (Siemens Healthineers, Forchheim, Germany; webviewcarbon version 4.51.2.0). Implant length and outer diameter were estimated preoperatively from the planned modified S1 corridor on multiplanar reconstructions, with a safety margin of 2–3 mm maintained ventrally toward the S1 promontory and superiorly–inferiorly toward the L5 and S1 nerve roots. Final implant dimensions were determined intraoperatively after guidewire placement on the basis of an intraoperative 3D scan, and implant length was measured using the manufacturer’s measuring gauge.

### 2.3. Contraindications and Dysmorphism Screening

Preoperative planning included systematic evaluation for sacral dysmorphism (anatomic variants of the upper sacrum). In patients with dysmorphic features such as a nonrecessed sacrum, mammillary bodies, or a tongue-in-groove configuration of the sacroiliac joint, the upper sacral segment often exhibits a narrowed osseous corridor. In such cases, a conventional transsacral corridor may be considered unsuitable because of the increased risk of L5 nerve root injury or violation of the S1 neuroforamen. In the present series, the modified cranial S1 trajectory was feasible in all patients, including those with sacral dysmorphism. Because the modified trajectory targets the cranial S1 promontory rather than a transsacral corridor, it is anatomically applicable in patients in whom conventional transverse trajectories are constrained by dysmorphic features.

### 2.4. Surgical Technique

The patient was positioned prone on a radiolucent carbon-fiber table to facilitate unobstructed fluoroscopic imaging. A C-arm fluoroscope was arranged to allow rotation between lateral, inlet, and outlet views. After the lumbosacral junction was identified, a true lateral view was obtained. A key fluoroscopic landmark for defining the modified entry point was superimposition of the greater sciatic notches, the posterior cortex of the S1 vertebral body, and the residual disc of S1/S2.

On the lateral view, the entry point was identified posterior and caudal to the standard S1 vestibular entry point used for transverse screw placement. After a small skin incision of approximately 20 mm, a 3.2-mm guidewire for the iFuse Implant System^®^ or iFuse TORQ^®^ implants, or a standard 3.2-mm guidewire for cannulated screws, was advanced to the iliac cortex. The guidewire was inserted manually or with a hammer rather than a power drill to provide tactile feedback during advancement and to differentiate cancellous resistance within the intended corridor from cortical resistance associated with the neural foramina or anterior cortex.

After the guidewire was seated in the ilium, the trajectory was verified using inlet and outlet fluoroscopic views. The wire was advanced medially, with an anterior direction on the inlet view and a cranial direction on the outlet view, toward the cranial S1 vertebral body. After fluoroscopic confirmation, an intraoperative 3D imaging, such as with a 3D C-arm, was performed in approximately 70% of procedures, with the institutional aim of intraoperative 3D verification in every case to exclude extraosseous placement or foraminal breach. After precise positioning had been confirmed, the tract was predrilled over the guidewire and the selected implant was inserted. Depending on the indication, sacroiliac stabilization was performed using either a cannulated screw or, when the sacroiliac joint was considered suitable, a 3D-printed titanium sacroiliac fusion implant (iFuse Implant System^®^ or iFuse TORQ^®^). Implant sizes used in the present series were as follows: 7.5-mm partially threaded titanium cannulated screws (length: 75–125 mm); iFuse Implant System^®^ triangular implants (7-mm side dimension; length: 35–90 mm); and iFuse TORQ^®^ cylindrical threaded implants (outer diameter: 10.0, 11.5, or 13.5 mm; length: 60–90 mm).

### 2.5. Technical Considerations

Accurate identification of the lateral fluoroscopic landmark—defined by superimposition of the greater sciatic notches—was critical for procedural reproducibility and safety. Deviating from this view altered the apparent safe corridor and increased the risk of anterior or posterior malposition. Manual advancement of the guidewire with a hammer provided tactile feedback during passage through cancellous bone and resistance upon contact with cortical structures, allowing for early recognition of potential cortical breaches. In cases of advanced osteoporosis, drilling was limited to opening the iliac cortex, with subsequent implant insertion used to compact cancellous bone rather than remove it, thereby creating a denser peri-implant bone bed.

### 2.6. Surgical Pearls and Pitfalls

Three major pitfalls were identified: superimposing the entry point on the lateral iliac surface and transferring it from preoperative planning to 2D fluoroscopy; angulating the guidewire toward the S1 promontory; and referencing the preoperatively measured implant length against the intraoperatively measured length.

**Figure 4:**
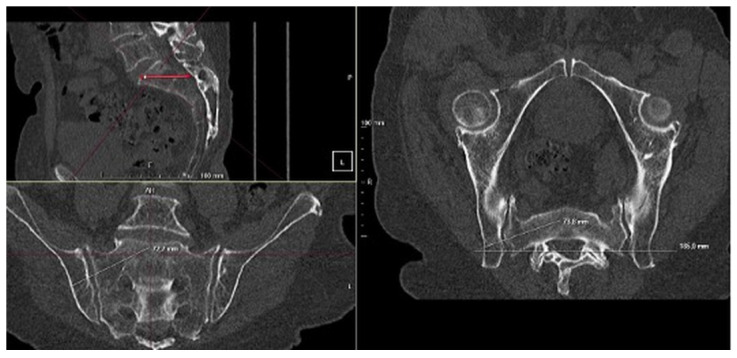
Preoperative CT-based planning and corridor measurement for the modified S1 trajectory. Multiplanar CT reconstructions (sagittal, coronal, and axial) are used to define the modified S1 corridor and determine the planned implant trajectory and length. The preoperatively measured implant length serves as an important intraoperative reference. In our practice, a discrepancy of approximately one implant-length increment, i.e., ≥5 mm from the planned length, prompts reassessment of the trajectory using lateral, inlet, and outlet fluoroscopic views, and intraoperative 3D imaging where available. A substantially shorter measured length may indicate that the trajectory is too steep, with risk of ventral sacral exit and potential L5 nerve conflict, whereas a substantially longer measured length may indicate that the trajectory is too flat, with risk of S1 neuroforaminal breach.

1. Identifying the correct entry point (often in the lateral view along the anterior or posterior border of the spinal canal) for the guidewire caudally and posteriorly;
2. Angulating the guidewire towards the S1 promontory, regularly crossing the Ala line;
3. Referencing the preoperatively measured length of the implant with the intraoperatively measured length (Figure 4). If the measured length is shorter than preoperatively planned length by ≥5 mm, it is most often because the trajectory angulation is too steep. Consequently, the wire does not pass into the promontory but remains lateral, possibly exiting the sacrum ventrally in the ala region, with a high risk of conflict with the L5 nerve root. If the measured length is longer than preoperatively planned length by ≥5 mm, the trajectory angulation is too flat and the guidewire has a high risk of crossing the S1 neuroforamen and extending onto the contralateral side of the sacrum. In this case, the promontory would not provide solid bone stock, even in the absence of associated neurologic risk.

### 2.7. Postoperative Management

Postoperative weight-bearing protocols were determined on the basis of the mechanism of injury. Patients with high-velocity injuries treated with cannulated screws were recommended partial weight-bearing activities. Patients with fragility fractures treated with 3D-printed titanium sacroiliac fusion implants were permitted weight-bearing activities as tolerated immediately after surgery to reduce complications related to immobility. Postoperative imaging with dual-energy CT was used to assess implant position and fracture reduction. Routine implant removal of screws was reserved for younger patients presenting with high-velocity injuries.

## 3. Results

### 3.1. Implant Distribution and Indications

**Figure 5:**
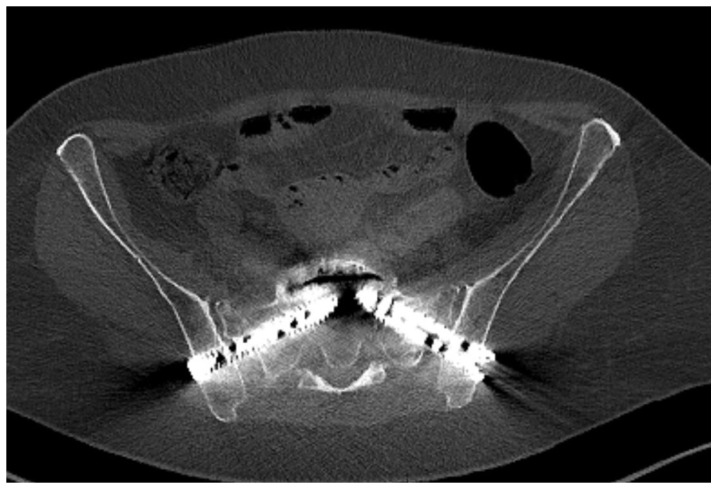
Postoperative axial CT verification of the modified S1 trajectory. The image demonstrates bilateral threaded cylindrical titanium fusion implants (13 mm diameter, 75 mm length) following the planned modified S1 corridor with complete intraosseous containment. The trajectory bypasses the sacral ala region and is directed toward the cranial-ventral S1 body, without evident cortical breach or foraminal encroachment.

A total of 137 implants were placed in 71 patients using the newly developed modified S1 corridor technique. Thirteen cannulated screws (9%) were placed in 8 patients with high-velocity pelvic ring injuries. With respect to the remaining 63 patients presenting with sacral insufficiency or low energy fractures, 72 standard 3D-printed triangular titanium sacroiliac fusion implants (iFuse Implant System^®^; 53%) were placed in 37 patients, and 52 threaded 3D-printed titanium sacroiliac fusion implants (iFuse TORQ^®^; 38%) were placed in 26 patients. A representative postoperative axial CT demonstrating the modified S1 trajectory with bilateral threaded cylindrical titanium fusion implants is shown in Figure 5. No patient received different implant types within the same procedure; each patient received a single implant type, allowing for unambiguous per-patient assignment to one of the three subgroups. The interval from injury to surgery differed systematically between subgroups: in patients with high-velocity pelvic ring trauma, surgical stabilisation was performed on the day of admission or on the following day, whereas in patients with FFPs, surgery was performed 5–7 days after a trial of conservative management.

### 3.2. Trajectory and Osseous Containment

In all cases, the preoperatively planned trajectory could be reproduced intraoperatively. The modified trajectory, originating from a posterior and caudal iliac entry point and directed anteriorly and cranially, consistently engaged the cranial S1 vertebral body. Postinsertion verification by postoperative CT in all 137 cases (per the institutional standard operating procedure), with intraoperative 3D imaging additionally used where available, demonstrated complete osseous containment of all 137 implants. No cortical breaches of the neuroforamina, spinal canal, sacral upper endplate or anterior sacral cortex were identified. No implant malposition was observed.

### 3.3. Clinical Safety and Revisions

No intraoperative complications related to guidewire advancement or implant insertion were observed. There were no documented iatrogenic nerve root (L5 or S1) or vascular injuries. Given the accuracy of fluoroscopic guidance combined with intraoperative 3D verification, no immediate intraoperative revisions were required. Follow-up imaging revealed maintenance of fracture reduction and implant position; implant removal was not necessary in any patient treated for fragility fractures. Follow-up for all patients was 12 ± 9 months (mean ± standard deviation). All patients underwent postoperative CT in accordance with the institutional standard operating procedure for the management of pelvic injuries, providing universal verification of implant position.

### 3.4. Implant Size in Comparison with Cannulated Screws

This novel surgical technique allows for the use of large fusion implants. Compared with cannulated screws, these implants provide a larger footprint and greater anchorage in osteoporotic bone, which may contribute to improved primary stability. The eight patients in the cannulated-screw subgroup, all with high-velocity pelvic ring injuries, had a mean age of 42 years (range: 28–60 years).

### 3.5. Potential Advantages of Fusion Implants over Cannulated Screws

In the present series, no loosening or backing-out was observed among any of the 137 implanted devices, and no implant required revision. However, this observation should be interpreted with caution, as cannulated screws were used exclusively in younger patients with high-velocity injuries, whereas fusion implants were primarily used in elderly patients with osteoporotic bone.

## 4. Discussion

Percutaneous iliosacral fixation is commonly used for stabilization of posterior pelvic ring injuries because of its minimally invasive nature and reduced soft tissue disruption [17]. The increasing incidence of FFPs necessitates reconsideration of standard fixation strategies in osteoporotic bone [17]. Conventional transverse screw placement, as reported by Krappinger et al., is effective in high-velocity trauma with preserved bone quality [4]; however, fluoroscopy-guided iliosacral screw placement remains technically demanding and has been associated with screw malposition rates of 2% to 15% and neurologic injury rates of 0.5% to 7.7%, making this trajectory less suitable in patients with dysmorphic sacral anatomies [18,19].

Using cannulated screws for insufficiency fractures of the osteoporotic sacrum is associated with complication rates of up to 30%, with screw loosening being the most common complication [4,20]. In the present series of patients, a new, density-oriented S1 trajectory was performed under standard 2D fluoroscopic guidance, with no observed implant misplacement. The implementation of 3D-printed titanium sacroiliac fusion implants—with nearly twice the diameter of cannulated screws—further supports the technical feasibility and short-term safety of this new technique.

The anatomical limitation of standard transverse fixation in geriatric patients is primarily related to bone quality and sacral dysmorphism. The conventional vestibular trajectory typically follows a strictly transverse path across the S1 vertebrae using cannulated screws, a transsacral bar, or a full-threaded screw spanning and crossing six cortices. Although appropriate in non-osteoporotic bone, this path typically traverses the sacral ala in elderly patients. Morphometric and densitometric analyses have identified the sacral ala as a region of low bone mineral density, frequently termed the “alar void” [2,3]. Fixation in this region provides limited toggling resistance and has been associated with early loosening and backing out in osteoporotic bone, specifically when cannulated screws are being used, even when these voids are being bridged by the screws [2,3]. Revision rates are as high as 20% in screw osteosynthesis for osteoporotic or fragility fractures of the sacrum [21].

The technique described in this study redirects the fixation target toward the cranial and ventral aspect of the S1 vertebral body, the promontory. Preoperative 3D planning is used to define a posterior and caudal iliac entry point, enabling an oblique cranial and anterior trajectory that bypasses the alar region. Quantitative analyses have shown that the cranial portion of the S1 vertebral body retains a comparatively higher bone mineral density, even in osteopenic pelves [2]. In the present series of patients, this trajectory consistently achieved osseous containment and stable implant positioning, without evidence of loosening during the observed follow-up.

In addition to trajectory selection, implant geometry affects fixation stability. Cannulated screws rely on thread anchorage and compression to maintain reduction. In osteoporotic bone, limited trabecular support may permit micromotion, commonly termed the “windshield wiper” effect, which can result in progressive instability, nonunion, screw loosening and screw backout. The 3D-printed titanium sacroiliac fusion implants presented in this study achieved an interference fit within cancellous bone. Biomechanical testing has shown that large-diameter threaded implants of this design achieve higher insertion and removal torques than standard 7.3 mm trauma lag screws [16]. These findings suggest increased resistance to loosening in osteoporotic bone where screw thread anchorage alone may be insufficient. In addition, porous surface technology has been associated with bone apposition and osseointegration, indicating long-term implant integration [22]. These characteristics are relevant in the management of fragility fractures, where early mobilization is often prioritized. Our results support these biomechanical findings regarding resistance to loosening.

Navigation-assisted techniques and robotic guidance have been shown to reduce rates of screw malposition; however, they remain costly and time-consuming and are not universally available [23]. A key observation in this study was that accurate implant placement could be achieved using standard 2D fluoroscopy when preoperative 3D planning is carefully translated to intraoperative fluoroscopic landmarks. Prone positioning was specifically selected for this technique as it provides direct posterior access to the caudal iliac entry point and facilitates fluoroscopic alignment of the greater sciatic notches in the lateral view. In particular, using a lateral view with superimposed greater sciatic notches facilitated reproducible identification of the entry point. Surgeons found manual advancement of the guidewire with a hammer helpful because it provided tactile feedback for detecting changes in resistance during advancement. Notably, a large number of implants were inserted without any neurological, neurovascular, or implant-related complications, with implant sizes and diameters far exceeding (≤7.5 mm) those of conventional 7.3 mm cannulated screws.

The absence of observed neurological, vascular, or implant-related complications in this series should be interpreted cautiously. Although these findings support the technical feasibility and short-term safety of the modified S1 corridor, they do not by themselves prove inherent superiority of the trajectory over conventional fixation methods. Several alternative explanations may have contributed to the low complication rate, including careful patient selection, exclusion of displaced fractures requiring open reduction or decompression, accumulated surgical experience with pelvic fixation, and systematic verification of the planned trajectory using preoperative 3D planning, multiplanar fluoroscopy, and intraoperative or postoperative 3D CT imaging. Therefore, the present findings should be regarded as proof of concept for accurate and reproducible implant placement rather than comparative evidence of clinical superiority.

This study has several limitations. The retrospective design and absence of a control group treated with standard transverse screws limit comparative conclusions. Although placement accuracy and short-term safety were verified, long-term clinical outcomes and comparative fixation durability were not assessed. Prospective studies are needed to elucidate functional outcomes and longer-term implant performance.

## 5. Conclusions

The modified S1 corridor technique is an adaptation of established posterior pelvic fixation strategies to the biomechanical conditions encountered in fragility fractures. By integrating density-based 3D planning into 2D fluoroscopic execution, this new approach employs 3D-printed titanium sacroiliac fusion implants to support accurate implant placement and stable fixation in osteoporotic bone. Compared with conventional transverse screw trajectories, this new approach provides an alternative when bone quality limits standard fixation. This modified S1 corridor can also be used for conventional cannulated screw placement.

## Data Availability

The data presented in this study are available upon request from the corresponding author.
